# The impact of racism on the future health of adults: protocol for a prospective cohort study

**DOI:** 10.1186/s12889-019-6664-x

**Published:** 2019-03-28

**Authors:** James Stanley, Ricci Harris, Donna Cormack, Andrew Waa, Richard Edwards

**Affiliations:** 1 Department of Public Health, University of Otago, Wellington, 23a Mein Street, Newtown, Wellington, New Zealand; 2 Eru Pōmare Māori Health Research Centre, University of Otago, Wellington, 23a Mein Street, Newtown, Wellington, New Zealand

**Keywords:** Racism, Prospective cohort study, Health service utilisation, Health inequities, New Zealand

## Abstract

**Background:**

Racial discrimination is recognised as a key social determinant of health and driver of racial/ethnic health inequities. Studies have shown that people exposed to racism have poorer health outcomes (particularly for mental health), alongside both reduced access to health care and poorer patient experiences. Most of these studies have used cross-sectional designs: this prospective cohort study (drawing on critical approaches to health research) should provide substantially stronger causal evidence regarding the impact of racism on subsequent health and health care outcomes.

**Methods:**

Participants are adults aged 15+ sampled from 2016/17 New Zealand Health Survey (NZHS) participants, sampled based on exposure to racism (ever exposed or never exposed, using five NZHS questions) and stratified by ethnic group (Māori, Pacific, Asian, European and Other). Target sample size is 1680 participants (half exposed, half unexposed) with follow-up survey timed for 12–24 months after baseline NZHS interview. All exposed participants are invited to participate, with unexposed participants selected using propensity score matching (propensity scores for exposure to racism, based on several major confounders). Respondents receive an initial invitation letter with choice of paper or web-based questionnaire. Those invitees not responding following reminders are contacted for computer-assisted telephone interview (CATI).

A brief questionnaire was developed covering current health status (mental and physical health measures) and recent health-service utilisation (unmet need and experiences with healthcare measures). Analysis will compare outcomes between those exposed and unexposed to racism, using regression models and inverse probability of treatment weights (IPTW) to account for the propensity score sampling process.

**Discussion:**

This study will add robust evidence on the causal links between experience of racism and subsequent health. The use of the NZHS as a baseline for a prospective study allows for the use of propensity score methods during the sampling phase as a novel approach to recruiting participants from the NZHS. This method allows for management of confounding at the sampling stage, while also reducing the need and cost of following up with all NZHS participants.

**Electronic supplementary material:**

The online version of this article (10.1186/s12889-019-6664-x) contains supplementary material, which is available to authorized users.

## Background

Differential access to the social determinants of health both creates and maintains unjust and avoidable health inequities [1]. In New Zealand, these inequities are largely patterned by ethnicity, particularly for Māori (the indigenous peoples) and Pacific peoples, and intertwined with ethnic distributions of socioeconomic status [2, 3]. In models of health, racism is recognised as a key social determinant that underpins systemic ethnic health and social inequities, as is evident in New Zealand and elsewhere [4, 5].

Racism can be understood as an organised system based on the categorisation and ranking of racial/ethnic groups into social hierarchies whereby ethnic groups are assigned differential value and have differential access to power, opportunities and resources, resulting in disadvantage for some groups and advantage for others [4, 6]. Historical power relationships underpin systems of racism [7], which in New Zealand relates specifically to our colonial history and ongoing colonial processes [8].

Racism can be expressed at structural and individual levels, with several taxonomies describing different levels of racism. Institutionalised racism, for example, has been defined as, “the structures, policies, practices, and norms resulting in differential access to the goods, services, and opportunities of society by race[/ethnicity]” (p. 10) [6]. In contrast, personally-mediated racism has been defined as, “prejudice and discrimination, where prejudice is differential assumptions about the abilities, motives, and intents of others by ‘race[/ethnicity],’ and discrimination is differential actions towards others by ‘race[/ethnicity]’” (p. 10) [6].

The multifarious expressions of racism can affect health via several recognised direct and indirect pathways. Indirect pathways include differential access to societal resources and health determinants by race/ethnicity, as evidenced by long-standing ethnic inequities in income, education, employment and living standards in New Zealand, with subsequent impacts on living environments and exposure to risk and protective factors [4, 6, 9, 10]. At the individual level, experience of racism can affect health directly through physical violence and stress pathways, with negative psychological and physiological impacts leading to subsequent mental and physical health consequences. In addition, racism influences healthcare via institutions and individual health providers, leading to ethnic inequities in access to and quality of care. For example, ethnic disparities in socioeconomic status can indirectly result in differential access to care, while health provider ethnic bias can influence the quality and outcomes of healthcare interactions [11].

There has been considerable recent growth in research supporting a direct link between experience of racism and health. A recent systematic review and meta-analysis summarised the evidence for direct links between self-reported personally-mediated racism and negative physical and mental health outcomes [12], with the strongest effect sizes demonstrated for mental health. Related work has also shown that experience of racial discrimination is associated with other adverse health outcomes and preclinical indicators of disease and health risk across various ethnic groups and countries, including in New Zealand [9, 13–15]. Experience of racism has also been linked to a range of negative health care-related measures [16].

However, most studies have used cross-sectional designs: very few of the articles in a recent systematic review [12] used prospective or longitudinal designs (*n* = 30, 9% of total, including multiple articles from some studies), limiting our ability to draw strong causal conclusions as the direction of causality cannot be determined when racism exposure and health outcomes are measured at the same time. Additionally, cross-sectional studies may give biased estimates of the magnitude of association between experience of racism and health: for example, bias may occur if experience of ill health (outcome) increases reporting or perception of racism (exposure) [12]. This is suggested by meta-analyses where effect sizes for the association between racism and mental health were larger for cross-sectional compared to longitudinal studies [12]. Longitudinal research on the effects of racism has been particularly limited with respect to physical health outcomes and measures of healthcare access and quality [12, 16]. Finally, existing prospective studies have largely been restricted to quite specific groups (e.g. adolescents, females, particular ethnic groups), with a limited number of studies undertaken at a national population level and few with sufficient data to explore the impact of racism on the health of Indigenous populations [12].

In New Zealand, reported experience of racism is substantially higher among Māori, Asian and Pacific ethnic groupings compared to European [3, 17]. In our own research, we have examined cross-sectional links between reported experience of racism and various measures of adult health in New Zealand using data from the New Zealand Health Survey (NZHS), an annual national survey by the Ministry of Health including ~ 13,000 adults per annum [2, 18, 19]. In these studies [17, 20–22] we have shown that both individual experience of racism (e.g. personal attacks or unfair treatment) and markers of structural racism (deprivation, other socioeconomic indicators) are independently associated with poor health (mental health, physical health, cardiovascular disease), health risks (smoking, hazardous alcohol consumption) and healthcare experience and use (screening, unmet need and negative patient experiences). Other New Zealand researchers have reported similar findings including studies among older Māori [23], adolescents [24], and for maternal and child health outcomes [25]. However, evidence from New Zealand prospective studies is still limited. The *NZ Attitudes and Values* study showed that, among Māori, experience of racism was negatively linked to subsequent wellbeing [26], and the *Growing Up in New Zealand* study reported that maternal experience of racism (measured antenatally) was linked to a higher risk of postnatal depression among Māori, Pacific and Asian women [27].

While empirical evidence of the links between racism and health is growing in New Zealand, it remains limited in several areas. There is consistent evidence from cross-sectional studies for the hypothesis that racism is associated with poorer health and health care. This study seeks to build on existing research to provide more robust causal evidence using a prospective design that helps to rule out reverse causality, in order to inform policy and healthcare interventions.

### Theoretical and conceptual approaches

Addressing racism as a health determinant is intrinsically linked to addressing ethnic health inequities. In New Zealand, Māori health is of special relevance given Māori rights under the Treaty of Waitangi [28] and the United Nations Declaration on the Rights of Indigenous People [29], and in recognition of the inequities for Māori across most major health indicators [28]. We recognise the direct significance of this project to Māori and understand racism in its broader sense as underpinning our colonial history with ongoing contemporary manifestations and effects [8]. As such, our work is informed by critical approaches to health research that are explicitly concerned with understanding inequity and transforming systems and structures to achieve the goal of health equity. This includes decolonising and transformative research principles [30] that influence our approach to the research question, data collection, analysis and interpretation of data, and translation of research findings. The team includes senior Māori researchers as well as advisors with experience in Māori health research and policy.

## Methods

### Aims and research questions

The overall aim is to examine the relationship between reported experience of racism and a range of subsequent health measures. The specific objectives are:

1. To determine whether experience of racism leads to poorer mental health and/or physical health.
2. To determine the impact of racism on subsequent use and experience of health services.

### Study design

The proposed study uses a prospective cohort study design. Respondents from the 2016/17 New Zealand Health Survey [2, 18, 19] (NZHS) provide the source of the follow-up cohort sample and the NZHS provides baseline data. The follow-up survey will be conducted between one and two years after respondents completed the NZHS. Using the NZHS data as our sampling frame provides access to exposure status (experience of racism), along with data on a substantial number of covariates (including age, gender, and socioeconomic variables) allowing us to select an appropriate study cohort for answering our research questions. Participant follow-up will be conducted by a multi-modality survey (mail, web and telephone modalities).

### Setting

This study explores the impact of racism on health in the general NZ adult population (which is the target population of the NZHS that forms the baseline of the study).

### Participants

Participants were selected from adult NZHS 2016/17 interviewees (*n* = 13,573, aged 15+ at NZHS interview) who consented to re-contact for future research within a 2 year re-contact window (92% of adult respondents). The NZHS is a complex-sample design survey with an 80% response rate for adults [18] and oversampling of Māori, Pacific, and Asian populations (who experience higher levels of racism), which facilitates studying the impact of racism on subsequent health status. Participants who had consented to re-contact (*n* = 12,530) also needed to have contact details recorded and sufficient data on exposures/confounders to be included in the sampling frame (*n* = 11,775, 93.9% of consenting adults). All invited participants will be aged at least 16 at the time of follow-up, as at least one year will have passed since participation in the NZHS (where all participants were aged at least 15).

Exposure to racism was determined from the five previously validated NZHS items [31] asked of all adult respondents (see Table 1) about personal experience of racism across five domains (verbal and physical attack; unfair treatment in health, housing, or work). Response options for each question cover recent exposure (within the past 12 months), more historical exposure (> 12 months ago), or no exposure to racism.

**Table 1:** Racism questions in the NZHS 2016/17 questionnaire [19]

Item #	Racism question:	Response options
1, 2	Have you ever been a victim of an ethnically motivated attack (verbal or physical abuse to you or your property) in New Zealand?	Yes, within the past 12 months Yes, more than 12 months ago No Don’t know Refused (n.b. items 1 and 2 are asked in a single question, but responses are recorded separately. Items 3–5 also have a ‘not applicable’ option) Respondents can record both recent (past 12 months) and historical exposure (more than 12 months ago)
3	Have you ever been treated unfairly (for example, kept waiting or treated differently) by a health professional (that is, a doctor, nurse, dentist etc) because of your ethnicity in New Zealand?	
4	Have you ever been treated unfairly at work or been refused a job because of your ethnicity in New Zealand?	
5	Have you ever been treated unfairly when renting or buying housing because of your ethnicity in New Zealand?	

### Identification of exposed and unexposed individuals

Individuals were classified as exposed to racism if they answered “yes” to any question in Table 1, in either timeframe (recent or historical: referred to as “ever” exposure). This allows for analysis restricted to the nested subset of individuals reporting recent exposure to racism (past 12 months) and those only reporting more historical exposure (> 12 months ago). The unexposed group comprised all individuals answering “No” to all five domains of experience of racism. We selected all exposed individuals for follow-up, along with a matched sample of unexposed individuals. Individuals missing exposure data were explicitly excluded.

### Matching of exposed and unexposed individuals

To address potential confounding, we used propensity score matching methods in our sampling stage to remove the impact of major confounders (as measured in the NZHS) of the causal association between experience of racism and health outcomes. Propensity score methods are increasingly used in observational epidemiology as a robust method for dealing with confounding in the analysis stage [32–36] and have more recently been considered as a useful approach for secondary sampling of participants from existing cohorts for subsequent follow up [37].

All exposed NZHS respondents will be invited into the follow-up survey. To find matched unexposed individuals, potential participants were stratified based on self-reported ethnicity (Māori, Pacific, Asian, European and Other; using prioritised ethnicity for individuals identifying with more than one grouping) [38] and then further matched for potential sociodemographic and socioeconomic confounders using propensity score methods [39, 40]. Stratification by ethnicity reflects the differential prevalence of racism by ethnic group, and furthermore allows ethnically-stratified estimates of the impact of racism [22].

Propensity scores were modelled using logistic regression for “ever” exposure to racism based on major confounder variables of the association between racism and poor health (Table 2), with modelling stratified by ethnic group. Selection of appropriate confounders was based on past work using cross-sectional analysis of the 2011/12 NZHS (e.g. [21, 22]) and the wider literature that informed the conceptual model for the project. Some additional variables were considered for inclusion in the matching process but were removed prior to finalisation (details in Table 2).

**Table 2:** List of confounders and covariates used in propensity score processing

Variable	Groups/levels	Comments
VARIABLES INCLUDED IN FINAL PROPENSITY SCORE MODEL		
Prioritised ethnicity	Māori Pacific Asian Other (Middle Eastern, Latin American, African, and Other) European/NZ European	Variable used to stratify groups prior to calculation of propensity scores
Nativity	Born in New Zealand Born outside New Zealand	
Age group	Ten-year age bands (15–24, 25–34, 35–44, 45–54, 55–64, 65–74, 75+)	Treated as categorical variable
Gender	Female Male	
NZDep2013 Quintile [54]	Quintiles 1–5	Treated as categorical variable
Highest educational qualification	Less than upper secondary Upper secondary Tertiary Other	“Other” category included qualification responses that could not be coded to an existing response category.
Employment status	Currently working Looking for work Not in labour force Not in labour force (aged 65+) Other	“Not in labour force” group split into those below and above retirement age (65+). “Other” category included responses that could not be coded to an existing response category.
VARIABLES CONSIDERED BUT NOT INCLUDED IN FINAL PROPENSITY SCORE MODEL		
Baseline self-rated health	Excellent/Very good/Good Fair/Poor	Health measure: not included as analysis adjusts for baseline
Baseline mental distress (K10 scale)	Continuous variable (score 0–40)	Health measure: not included as analysis adjusts for baseline
Smoking status	Current smoker (at least monthly) Not current smoker	Health measure: not included (also substantial missing data)
Index of Multiple Deprivation [55] (IMD)	Decile for total IMD score	Overlap with NZDep quintile; did not improve balance
IMD [55] Employment Subscale	Decile for employment measures in local area	Overlap with NZDep quintile (individual level employment also already included)

Within each ethnic group stratum, exposed individuals were matched with unexposed individuals (1:1 matching) based on propensity scores to make these two groups approximately exchangeable (confounders balanced between exposure groups). The matching process [41] used nearest neighbour matching as implemented in MatchIt [42] in R 3.4 (R Institute, Vienna, Austria). As the propensity score modelling is blind to participants’ future outcome status, the final propensity score models were refined using just the baseline NZHS data to achieve maximal balance of confounders between exposure groups, without risking bias to the subsequent primary causal analyses [39]. Balance between groups was then checked on all matching variables prior to finalisation of the sampling lists.

### Questionnaire development

**Fig. 1:**
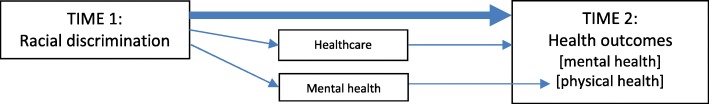
Potential pathways between racism and health outcomes. Direct pathway: Main arrow represents the direct biopsychosocial and trauma pathways between experience of racial discrimination (Time 1) and negative health outcomes (Time 2) Indirect pathways: Racial discrimination (Time 1) can impact negatively on health outcomes (Time 2) via healthcare pathways (e.g. less engagement, unmet need). Racial discrimination (Time 1) can impact negatively on physical health outcomes (Time 2) via mental health pathways

**Fig. 2:**
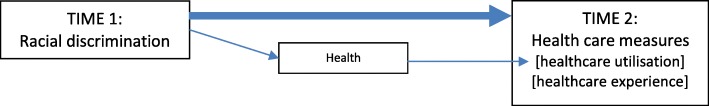
Potential pathways between racism and healthcare utilisation outcomes. Main pathway: Main arrow represents the pathway between experience of racial discrimination (Time 1) and negative healthcare measures (Time 2), via negative perceptions and expectations of healthcare (providers, organisations, systems) and future engagement. Secondary pathway: Racial discrimination (T1) can impact negatively on healthcare (Time 2) via negative impacts on health increasing healthcare need

Development of the follow-up questionnaire was informed by a literature review and a conceptual model (Figs. 1 and 2) of the potential pathways from racism to health outcomes (Fig. 1) and health service utilisation (Fig. 2) [4, 10, 16, 43, 44]. The literature review focussed on longitudinal studies of racism and health among adolescents and adults that included health or health service outcomes. The literature review covered longitudinal studies post-dating the 2015 systematic review by Paradies et al. [12], using similar search terms for papers between 2013 and 2017 indexed in Medline and PubMed databases, alongside additional studies from systematic reviews [12, 16].

We used several criteria for considering and prioritising variables for the questionnaire. The conceptual model also informed prioritisation of variables for the questionnaire. For outcome measures, these included: alignment with study aims and objectives; existing evidence of a relationship between racism and outcome; New Zealand evidence of ethnic inequities in outcome; previous cross-sectional relationships between racism and outcome in New Zealand data; availability of baseline measures (for health outcomes); plausibility of health effects manifesting within a 1–2 year follow-up period; and data quality (e.g. validated measures, low missing data, questions suitable for multimodal administration). Mediators and confounders were considered for variables not available in the baseline NZHS survey, as was recent experience of racism (following the NZHS interview) to provide additional measurement of exposure to recent racism. A final consideration for prioritising items for inclusion was keeping the length of the questionnaire short in order to maximise response rates (while being able to fully address the study aims). The questionnaire was extensively discussed by the research team and reviewed by the study advisors prior to finalisation.

### Outcomes

Table 3 summarises the outcome measures by topic domain and original source (with references). The final questionnaire content can be found in the Additional file 1, and includes: health outcome measures of mental and physical health (using SF12-v2 and K10 scales); health service measures (unmet need, satisfaction with usual medical centre, experiences with general practitioners); experience of racism in the last 12 months (adapted from items in the NZHS); and variables required to restrict data (e.g. having a usual medical centre, type of centre, having a General Practitioner [GP] visit in the last 12 months) or potential confounder and mediator variables not available at baseline (e.g. number of GP visits).

**Table 3:** Topics covered in questionnaire, with outcome variables

Topic	Outcome variable (Q# in questionnaire)	Notes	Sources and references
Mental health	Kessler 10 (K10) psychological distress in last 4 weeks (Q9) SF-12v2 Mental Health sub-scale (Q1–7)	SF-12v2 not included in Additional file 1 (copyrighted)	2016/17 NZHS [19, 56–58]
Physical health	SF-12v2 Physical Health sub-scale(Q1–7)	SF-12v2 not included in Additional file 1 (copyrighted)	2016/17 NZHS [19, 57, 58]
Self-rated health	First item of SF-36/SF-12 (Q1)		2016/17 NZHS [19]
Unmet health care need	Any unmet health care need (Q8) Any unmet need for mental health, including substance abuse (Q10) Any unmet need for General Practitioner (GP) visit (Q20, 21)	All cover last 12 months	Australian GSS [59, 60] 2016/17 NZHS [19] 2011/12 NZHS [61]
Health care centre	Has a usual health care centre (Q11) Type of usual health care centre (Q12) Satisfaction with usual medical centre (Q13)	Satisfaction asked for last 12 months (if visited usual centre)	2016/17 NZHS [19] 2011/12 NZHS [61]
Health care experience	Saw GP in last 12 months for own health (Q14) Number of times saw GP last 12 months (Q15) Experience with GP on last visit (Q16–19): Explained conditions/treatment (Q16) Involved patient in decisions (Q17) Treated with respect & dignity (Q18) Has confidence and trust in GP (Q19)		2016/17 NZHS [19] 2011/12 NZHS [61]
Recent experience of racial discrimination	See NZHS questions in Table 1 (Q22–25)	Adapted NZHS questions (Table 1) for last 12 months	2016/17 NZHS [19]

### Recruitment and data collection

Recruitment is currently underway. The sampling phase provided a list of potential participants for invitation, and recruitment for the follow-up survey uses the contact details from the NZHS interview (physical address, mobile/landline telephone, and email address if available). Recruitment will take place over three tranches to (1) manage fieldwork capacity and (2) allow tracking of response rates and adaptation of contact strategies if recruitment is sub-optimal.

To maximise response rates, we chose to use a multi-modal survey [45]. Participants are invited to respond by a paper questionnaire included with the initial invitation letter (questionnaire returned by pre-paid post), by self-completed online questionnaire, or by computer-assisted telephone interview (CATI, on mobile or landline.) A pen is included in the study invitation to improve initial engagement with the paper-based survey [46]. Participants completing the survey are offered a NZ$20 gift card to recognise their participation. The contact information contains instructions for opting out of the study.

Those participants not responding online or by post receive a reminder postcard mailed out two weeks after the initial letter, containing a link to the web survey and a note that the participant will be contacted by telephone in two weeks’ time.

Two weeks after the reminder postcard (four weeks post-invitation) remaining non-respondents are contacted using CATI processes. For those with mobile phone numbers or email addresses, a text (SMS) or email reminder is sent two days before the telephone contact phase. Once contact is made by telephone, the interviewer asks the participant to complete the survey over the telephone at that time or organises a subsequent appointment (interview duration approximately 15 min). Interviewers make up to seven telephone contact attempts for each participant, using all recorded telephone numbers. Respondents who decline to complete the full interview at telephone follow-up are asked to consider answering two priority questions (self-rated health and any unmet need for healthcare in the last 12 months: questions 1 and 8 in Table 3 and Additional file 1).

Past surveys conducted in NZ have frequently noted lower response rates and hence under-representation of Māori [47, 48]. Drawing on Kaupapa Māori research principles, we are explicitly aiming for equitable response rates of Māori to ensure maximum power for ethnically stratified analysis. This involves providing culturally appropriate invitations and interviewers for participants, and actively monitoring response rates by ethnicity during data collection to allow longer and more frequent follow-up of Māori, Pacific and Asian participants if required [48, 49]. The use of a multi-modal survey is also expected to minimise recruitment problems inherent to any single modality (e.g. lower phone ownership or internet access in some ethnic groups).

We have contracted an external research company to co-ordinate recruitment and data collection fieldwork under our supervision (covering all contact processes described here), which follows recruitment and data management protocols set by our research team.

### Statistical analysis

Propensity score methods for the sampling stage are described above: this section focuses on causal analyses for health outcomes in the achieved sample. The sampling frame selects participants based on “ever” experience of racism, which is our exposure definition.

All analyses will account for both the complex survey sampling frame (weights, strata and clusters from the NZHS) and the secondary sampling phase (selection based on propensity scores). Complex survey data will be handled using software to account for these designs (e.g. survey package [50] in R); propensity scores will be handled in the main analysis by using inverse probability of treatment weights (IPTW) combined with the sampling weights [51].

Linear regression methods will be used to compare change in continuous outcome measures (e.g. K10 score) by estimating mean score at follow-up, adjusted for baseline. Analysis of dichotomous categorical outcomes (e.g. self-rated health) will use logistic regression methods, again adjusted for baseline (for health outcomes). We will conduct analyses stratified by ethnic group to explore whether the impact of racism differs by ethnic group. Models will adjust for confounders included in creating the propensity scores (doubly-robust estimation) to address residual confounding not fully covered by the propensity score approach [52]. Analysis for other outcomes will use similar methods.

As we hypothesise that some outcomes (e.g. self-reported mental distress) will be more strongly influenced by recent experience of racism, we will also examine our main outcomes restricted to those only reporting historical (more than 12 months ago) or recent (last 12 months) racism at baseline. These historical and recent experience groups (and corresponding unexposed individuals) form nested sub-groups of the total cohort, and so analysis will follow the same framework outlined above. Experience of racism in the last 12 months (measured at follow-up) will be examined in cross-sectional analyses and in combination with baseline measures of racism to create a measure to examine the cumulative impact of racism on outcomes.

### Sensitivity analyses

While the sampling invitation lists are based on matched samples, we have no control about specific individuals choosing to participate in the follow-up survey, and so the original matching is unlikely to be maintained in the achieved sample. We will conduct sensitivity analyses using re-matched data (based on propensity scores for those participating in follow-up) to allow for re-calibration of exposed and unexposed groups in the achieved sample.

To consider potential for bias due to non-response in our follow-up sample, we will compare NZHS 2016/17 cross-sectional data for responders and non-responders on baseline sociodemographic, socioeconomic, and baseline health variables.

### Sample size

Based on NZHS 2011/12 responses, we anticipated a total pool of 2100 potential participants with “ever” experience of racism, with approximately 1100 expected to be Māori/Pacific/Asian ethnicity, and 10,000 with no report of racism (at least 2 unexposed per exposed individual in each ethnic group).

For the main analyses (based on “ever” experience of racism) we assumed a conservative follow-up rate of 40%, giving a final sample size of at least 840 exposed individuals. This response rate includes re-contact and agreement to participate, based on past experience recruiting NZHS participants for other studies and the relative length of the current survey questionnaire.

Initial projections (based on NZHS2011/12 data) indicated sufficient numbers of unexposed individuals for 1:1 matching based on ethnicity and propensity scores. This gives a feasible total sample size of *n* = 1680, providing substantial power for the K10 mental health outcome (standard deviation = 6.5: > 95% power to detect difference in change of 2 units of K10 between groups.) For the second main health outcome (change in self-rated health), this sample size will have > 85% power for a difference between 8% of those exposed to racism having worse self-reported health at follow-up (relative to baseline) compared to 5% of unexposed individuals.

For analyses of effects stratified by ethnicity, we expect > 95% power for Māori participants (*n* = 280 each exposed and unexposed) for the K10 outcome (assumptions as above); change in self-rated health will have 80% power for a difference between 12% of exposed individuals having worse self-reported health at follow-up (relative to baseline) compared to 5% of unexposed individuals. Stratified estimates for Pacific and Asian groups will have poorer precision, but should still provide valid comparisons.

### Ethical approval and consent to participate

The study involves recruiting participants who have already completed the NZHS interview (including questions on racial discrimination) The NZHS as conducted by the Ministry of Health has its own ethical approval (MEC/10/10/103) and participants are only invited onto the present study if they explicitly consented (at the time of completing the NZHS) to re-contact for future health research. The current study was reviewed and approved by the University of Otago’s Human Ethics (Health) Committee prior to commencement of fieldwork (reference: H17/094). Participants provided informed consent to participate at the time of completing the follow-up survey depending on response modality: implicitly through completion and return of the paper survey which stated “By completing this survey, you indicate that you understand the research and are willing to participate” (see Additional file 1: a separate written consent document was not required by the ethics committee); in the online survey by responding “yes” to a similarly worded question that they understood the study and agreed to take part (recorded as part of data collection, and participation could not continue unless ticked), or by verbal consent in a similar initial question in the telephone interview (since written consent could not be collected in this setting). These consent methods were approved by the reviewing Ethics committee [53]. Ethical approval for the study included using the same consent processes for those participants aged 16 to 18 as for older participants.

## Discussion

This study will contribute robust evidence to the limited national and international literature from prospective studies on the causal links between experience of racism and subsequent health. The use of the NZHS as the baseline for the prospective study capitalises on the inclusion of racism questions in that survey to provide a unique and important opportunity to build on and substantially strengthen the current evidence base for the impact of racism on health using data spanning the entire New Zealand adult population. In addition, our use of propensity scores in the sampling phase is a novel approach to prospective recruitment of participants from the NZHS. This approach should manage confounding while reducing the need (and cost) of following up all NZHS participants, without compromising the internal validity of the results. The novel methods developed for using the NZHS as the base for a prospective cohort study will have wider application to other health priority areas. One general limitation of this approach is that baseline data (for both propensity score development and baseline health measures) is limited to the data captured in the existing larger survey. We anticipate that this study will assist in prioritising racism as a health determinant and inform the development of anti-racism interventions in health service delivery and policy making.

### Current stage of research

Funding for this project began October 1st 2017. The first set of respondent invitations was mailed out on July 12th 2018; fieldwork for the final tranche of invitations was underway at the time of submission and is expected to be completed by 31 December 2018. Analysis and reporting will take place in mid-to-late 2019.

## Additional file

Additional file 1: Questionnaire used in follow-up survey. (PDF 919 kb)

## Abbreviations

CATI: Computer Assisted Telephone Interview
GP: General Practitioner
GSS: General Social Survey
IMD: Index of Multiple Deprivation
IPTW: Inverse Probability of Treatment Weights
K10: Kessler 10
NZ: New Zealand
NZDep: New Zealand Deprivation Index
NZHS: New Zealand Health Survey
SF-12/SF-36: 12/36-Item Short Form Survey
SMS: short message service

## References

[1] Commission on Social Determinants of Health (2008). Closing the gap in a generation: health equity through actions on the social determinants of health.

[2] Ministry of Health. Annual Update of Key Results 2016/17: New Zealand Health Survey 2017 [Available from: https://www.health.govt.nz/publication/annual-update-key-results-2016-17-new-zealand-health-survey. accessed 13/09/2018].

[3] Ministry of Social Development (2016). The Social Report 2016: Te pūrongo oranga tangata.

[4] Williams DR, Mohammed SA. Racism and health I: pathways and scientific evidence. Am Behav Sci. 2013;57(8). 10.1177/0002764213487340. 10.1177/0002764213487340 PMC3863357 24347666

[5] Reid P, Robson B, Robson B, Harris R (2007). Understanding health inequities. Hauora: Maori standards of health IV a study of the years 2000–2005.

[6] Jones C (2002). Confronting institutionalized racism. Phylon.

[7] Garner S (2010). Racisms: An introduction.

[8] Becares L, Cormack D, Harris R (2013). Ethnic density and area deprivation: neighbourhood effects on Maori health and racial discrimination in Aotearoa/New Zealand. Soc Sci Med.

[9] Paradies YC (2006). Defining, conceptualizing and characterizing racism in health research. Crit Public Health.

[10] Krieger N (2012). Methods for the scientific study of discrimination and health: an ecosocial approach. Am J Public Health.

[11] Jones CP (2001). Invited commentary: "race," racism, and the practice of epidemiology. Am J Epidemiol.

[12] Paradies Y, Ben J, Denson N (2015). Racism as a determinant of health: a systematic review and meta-analysis. PLoS One.

[13] Lewis TT, Cogburn CD, Williams DR (2015). Self-reported experiences of discrimination and health: scientific advances, ongoing controversies, and emerging issues. Annu Rev Clin Psychol.

[14] Gee GC, Ro A, Shariff-Marco S (2009). Racial discrimination and health among Asian Americans: evidence, assessment, and directions for future research. Epidemiol Rev.

[15] Williams DR, Mohammed SA (2009). Discrimination and racial disparities in health: evidence and needed research. J Behav Med.

[16] Ben J, Cormack D, Harris R (2017). Racism and health service utilisation: a systematic review and meta-analysis. PLoS One.

[17] Harris R, Cormack D, Tobias M (2012). The pervasive effects of racism: experiences of racial discrimination in New Zealand over time and associations with multiple health domains. Soc Sci Med.

[18] Ministry of Health. Methodology report 2016/17: New Zealand health survey. Wellington: Ministry of Health; 2017.

[19] Ministry of Health. Content guide 2016/17: New Zealand health survey. Wellington: Ministry of Health; 2017.

[20] Harris R, Cormack D, Tobias M (2012). Self-reported experience of racial discrimination and health care use in New Zealand: results from the 2006/07 New Zealand health survey. Am J Public Health.

[21] Harris RB, Cormack DM, Stanley J. Experience of racism and associations with unmet need and healthcare satisfaction: the 2011/12 adult New Zealand health survey. Aust N Z J Public Health. 2018. 10.1111/1753-6405.12835 published Online First: 2018/10/09. 10.1111/1753-6405.12835 30296819

[22] Harris RB, Stanley J, Cormack DM (2018). Racism and health in New Zealand: prevalence over time and associations between recent experience of racism and health and wellbeing measures using national survey data. PLoS One.

[23] Dyall L, Kepa M, Teh R (2014). Cultural and social factors and quality of life of Maori in advanced age. Te puawaitanga o nga tapuwae kia ora tonu - life and living in advanced age: a cohort study in New Zealand (LiLACS NZ). N Z Med J.

[24] Crengle S, Robinson E, Ameratunga S (2012). Ethnic discrimination prevalence and associations with health outcomes: data from a nationally representative cross-sectional survey of secondary school students in New Zealand. BMC Public Health.

[25] Thayer ZM, Kuzawa CW (2015). Ethnic discrimination predicts poor self-rated health and cortisol in pregnancy: insights from New Zealand. Soc Sci Med.

[26] Stronge S, Sengupta NK, Barlow FK (2016). Perceived discrimination predicts increased support for political rights and life satisfaction mediated by ethnic identity: a longitudinal analysis. Cult Divers Ethn Minor Psychol.

[27] Becares L, Atatoa-Carr P (2016). The association between maternal and partner experienced racial discrimination and prenatal perceived stress, prenatal and postnatal depression: findings from the growing up in New Zealand cohort study. Int J Equity Health.

[28] Robson B, Harris R (2007). Hauora : Maori standards of health IV : a study of the years 2000–2005.

[29] UN General Assembly. United Nations Declaration on the Rights of Indigenous Peoples : resolution / adopted by the General Assembly 2007. Available from: https://undocs.org/A/RES/61/295. Accessed 1 Nov 2018.

[30] Smith L (2012). Decolonizing methodologies: research and indigenous peoples.

[31] Harris R, Tobias M, Jeffreys M (2006). Racism and health: the relationship between experience of racial discrimination and health in New Zealand. Soc Sci Med.

[32] Sturmer T, Joshi M, Glynn RJ (2006). A review of the application of propensity score methods yielded increasing use, advantages in specific settings, but not substantially different estimates compared with conventional multivariable methods. J Clin Epidemiol.

[33] Luo Z, Gardiner JC, Bradley CJ (2010). Applying propensity score methods in medical research: pitfalls and prospects. Med Care Res Rev.

[34] Weitzen S, Lapane KL, Toledano AY (2004). Principles for modeling propensity scores in medical research: a systematic literature review. Pharmacoepidemiol Drug Saf.

[35] Shah BR, Laupacis A, Hux JE (2005). Propensity score methods gave similar results to traditional regression modeling in observational studies: a systematic review. J Clin Epidemiol.

[36] Stuart EA (2010). Matching methods for causal inference: a review and a look forward. Stat Sci.

[37] Stuart EA, Ialongo NS (2010). Matching methods for selection of subjects for follow-up. Multivariate Behav Res.

[38] Ministry of Health. Ethnicity Data Protocols Wellington: Ministry of Health; 2017. Available from: https://www.health.govt.nz/system/files/documents/publications/hiso-10001-2017-ethnicity-data-protocols-v2.pdf. Accessed 1 Nov 2018.

[39] Austin PC (2011). An introduction to propensity score methods for reducing the effects of confounding in observational studies. Multivariate Behav Res.

[40] Caliendo M, Kopeinig S (2008). Some practical guidance for the implementation of propensity score matching. J Econ Surv.

[41] Ho DE, Imai K, King G (2017). Matching as nonparametric preprocessing for reducing model dependence in parametric causal inference. Polit Anal.

[42] Ho DE, Imai K, King G, et al. MatchIt: nonparametric preprocessing for parametric causal inference. J Stat Softw. 2011;42(8):1–28.

[43] Sarnyai Z, Berger M, Jawan I (2016). Allostatic load mediates the impact of stress and trauma on physical and mental health in indigenous Australians. Australas Psychiatry.

[44] Kaholokula J (2015). Mauli Ola: Pathways toward Social Justice for Native Hawaiians.

[45] Fowler FJ, Roman AM, Mahmood R (2016). Reducing nonresponse and nonresponse error in a telephone survey: an informative case study. J Survey Stat Methodol.

[46] White E, Carney PA, Kolar AS (2005). Increasing response to mailed questionnaires by including a pencil/pen. Am J Epidemiol.

[47] Fink JW, Paine SJ, Gander PH (2011). Changing response rates from Maori and non-Maori in national sleep health surveys. N Z Med J.

[48] Paine SJ, Priston M, Signal TL (2013). Developing new approaches for the recruitment and retention of Indigenous participants in longitudinal research: Lessons from E Moe, Māmā: Maternal Sleep and Health in Aotearoa/New Zealand. MAI Journal: A New Zealand Journal of Indigenous Scholarship.

[49] Selak V, Crengle S, Elley CR (2013). Recruiting equal numbers of indigenous and non-indigenous participants to a 'polypill' randomized trial. Int J Equity Health.

[50] Lumley T. survey: analysis of complex survey samples v3.32 2017 [R package]. Available from: https://cran.r-project.org/web/packages/survey/index.html. Accessed 1 Nov 2018.

[51] Lenis D, Nguyen TQ, Dong N, et al. It's all about balance: propensity score matching in the context of complex survey data. Biostatistics. 2017. 10.1093/biostatistics/kxx063 [published Online First: 2018/01/03]. 10.1093/biostatistics/kxx063 PMC6296318 29293896

[52] Funk MJ, Westreich D, Wiesen C (2011). Doubly robust estimation of causal effects. Am J Epidemiol.

[53] National Ethics Advisory Committee (2012). Ethical guidelines for observational studies: observational research, audits and related activities. Revised edition.

[54] Atkinson J, Salmond C, Crampton P (2014). NZDep2013 index of deprivation.

[55] Exeter DJ, Zhao J, Crengle S (2017). The New Zealand indices of multiple deprivation (IMD): a new suite of indicators for social and health research in Aotearoa, New Zealand. PLoS One.

[56] Kessler RC, Andrews G, Colpe LJ (2002). Short screening scales to monitor population prevalences and trends in non-specific psychological distress. Psychol Med.

[57] Ware J, Kosinski M, Keller SD (1996). A 12-item short-form health survey: construction of scales and preliminary tests of reliability and validity. Med Care.

[58] Fleishman JA, Selim AJ, Kazis LE (2010). Deriving SF-12v2 physical and mental health summary scores: a comparison of different scoring algorithms. Qual Life Res.

[59] Bastos JL, Harnois CE, Paradies YC (2018). Health care barriers, racism, and intersectionality in Australia. Soc Sci Med.

[60] Australian Bureau of Statistics (2014). General Social Survey 2014: Household questionnaire.

[61] Ministry of Health. The New Zealand Health Survey: Content Guide and questionnaires 2011–2012 Wellington: Ministry of Health; 2012 [Available from: http://www.health.govt.nz/publication/new-zealand-health-survey-content-guide-and-questionnaires-2011-2012. accessed 31/10/2016 2016].

