# Supplementary material for: The impact of racism on the future health of adults: protocol for a prospective cohort study
Source: BMC Public Health. 2019 Mar 28;19:346. doi: 10.1186/s12889-019-6664-x (PMC6437906; doi:10.1186/s12889-019-6664-x)
Supplement: Supplementary file 1 — Questionnaire used in follow-up survey. (PDF 919 kb) [file 12889_2019_6664_MOESM1_ESM.pdf]

Health Survey

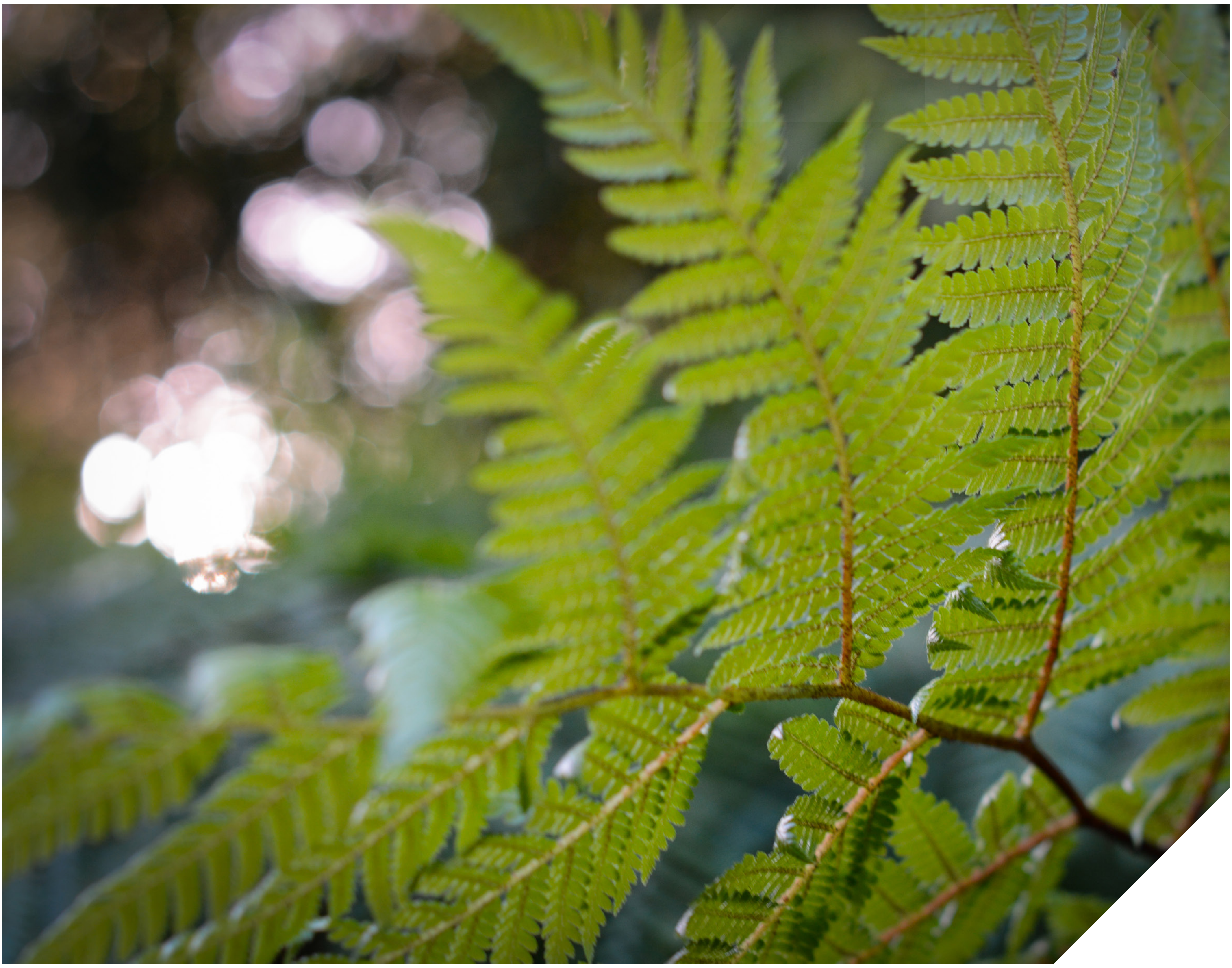

Research ID:

Health Survey

- Thank you for agreeing to complete this survey.
- Please only complete this survey if you are the named person on the accompanying letter.
- Please do NOT give the survey to anyone else to answer.
- By completing this survey, you indicate that you understand the research and are willing to participate.
- All responses will be treated as strictly confidential.
- Please clearly mark your answers using a tick in the corresponding box. ☑

Section 1: Your health now

1 In general, would you say your health is:

Excellent	Very Good	Good	Fair	Poor
☐	☐	☐	☐	☐

2 Q2-Q7 are copyrighted and not reproduced here.

SF-12v2® Health Survey © 1994, 2003, 2010 Health Assessment Lab, Medical Outcomes Trust and QualityMetric Incorporated

Section 2: Your physical and emotional health over the last 4 weeks

3 Q2-Q7 are copyrighted and not reproduced here.

SF-12v2® Health Survey © 1994, 2003, 2010 Health Assessment Lab, Medical Outcomes Trust and QualityMetric Incorporated

Q1-Q7 are from SF-12v2® Health Survey © 1994, 2003, 2010 Health Assessment Lab, Medical Outcomes Trust and QualityMetric Incorporated. All rights reserved. SF-12® is a registered trademark of Medical Outcomes Trust.

4 Q2-Q7 are copyrighted and not reproduced here.

SF-12v2® Health Survey © 1994, 2003, 2010 Health Assessment Lab, Medical Outcomes Trust and QualityMetric Incorporated

5 Q2-Q7 are copyrighted and not reproduced here.

SF-12v2® Health Survey © 1994, 2003, 2010 Health Assessment Lab, Medical Outcomes Trust and QualityMetric Incorporated

6 Q2-Q7 are copyrighted and not reproduced here.

SF-12v2® Health Survey © 1994, 2003, 2010 Health Assessment Lab, Medical Outcomes Trust and QualityMetric Incorporated

7 Q2-Q7 are copyrighted and not reproduced here.

SF-12v2® Health Survey © 1994, 2003, 2010 Health Assessment Lab, Medical Outcomes Trust and QualityMetric Incorporated

Q1-Q7 are from SF-12v2® Health Survey © 1994, 2003, 2010 Health Assessment Lab, Medical Outcomes Trust and QualityMetric Incorporated. All rights reserved. SF-12® is a registered trademark of Medical Outcomes Trust.

Section 3: Your use of health care in New Zealand

Research ID:

8 In the last 12 months, was there ever a time that you needed health care but could not get it?

- Yes ☐
- No ☐
- Not applicable – I did not need health care in the last 12 months ☐

Section 4. How you have been feeling

9 These next questions ask about how you have been feeling during the past 4 weeks. Some of these questions are similar to earlier questions, but we need to ask them again.

For each question, please select the option that best describes how often you had this feeling.

During the past 4 weeks, how often did you feel…

	All of the time	Most of the time	Some of the time	A little of the time	None of the time
…tired out for no good reason?	☐	☐	☐	☐	☐
…nervous?	☐	☐	☐	☐	☐
…so nervous that nothing could calm you down?	☐	☐	☐	☐	☐
…hopeless?	☐	☐	☐	☐	☐
…restless or fidgety?	☐	☐	☐	☐	☐
…so restless you could not sit still?	☐	☐	☐	☐	☐
…depressed?	☐	☐	☐	☐	☐
…so depressed that nothing could cheer you up?	☐	☐	☐	☐	☐
…that everything was an effort?	☐	☐	☐	☐	☐
…worthless?	☐	☐	☐	☐	☐

10 In the past 12 months, did you ever feel that you needed professional help for your emotions, stress, mental health, or substance use, but you didn't receive that help?

This could have been because of personal reasons (for example it cost too much) or reasons you couldn't control (for example no appointments available).

Substance use here means use of alcohol or drugs. Please do not include tobacco or nicotine products (e.g. cigarettes, other tobacco products, vapes).

- Yes ☐
- No ☐

11 Do you have a GP clinic or medical centre that you usually go to when you are feeling unwell or are injured?

This can include Student/Youth health services, Māori or Pacific health clinics, and Accident and Medical Centres.

- Yes ☐ → **GO TO Q12 (NEXT QUESTION)**
- No ☐ → **GO TO Q14 (NEXT PAGE)**

12 What sort of health care service is this?

If you go to two or more places, please only select the place you "usually" go to.

If you usually go to a Māori or Pacific health clinic, please select "GP clinic" from the list.
If you usually go to the Emergency Department (ED), please write this in the "Other" box.

- A GP clinic, medical centre or family practice ☐
- A student health service ☐
- A clinic that is after-hours or an Accident and Medical Centre – not an Emergency Department at a public hospital ☐
- Other – please write in: ________

We will call this place your usual medical centre.

13 Overall, how satisfied are you with the care you got at your usual medical centre in the last 12 months? This includes all staff not just the GP.

Very satisfied	Satisfied	Neither satisfied or dissatisfied	Dissatisfied	Very dissatisfied	Not applicable – I have not been to my usual medical centre in the last 12 months
☐	☐	☐	☐	☐	☐

14 In the past 12 months, have you seen a GP, or been visited by a GP, about your own health? By health, we mean your mental and emotional health as well as your physical health.

- Yes ☐ → GO TO Q15 (NEXT QUESTION)
- No ☐ → GO TO Q20 (NEXT PAGE)

15 How many times did you see a GP in the past 12 months?

Number of times ☐

16 Thinking about your last visit to a GP…
How good was the doctor at explaining your health conditions and treatments in a way that you could understand?

Very good	Good	Neither good or bad	Poor	Very Poor	Doesn't apply to last visit
☐	☐	☐	☐	☐	☐

17 Thinking about your last visit to a GP…
How good was the doctor at involving you in decisions about your care, such as discussing different treatment options?

Very good	Good	Neither good or bad	Poor	Very Poor	Doesn't apply to last visit
☐	☐	☐	☐	☐	☐

18 Thinking about your last visit to a GP…
How good was the doctor at treating you with respect and dignity?

Very good	Good	Neither good or bad	Poor	Very Poor	Doesn't apply to last visit
☐	☐	☐	☐	☐	☐

19 Still thinking about your last visit to a GP…
Did you have confidence and trust in the last GP you saw?

- Yes, definitely ☐
- Yes, to some extent ☐
- No, not at all ☐

20 In the last 12 months, has there been any time when you needed to see a GP about your own health, but didn't get to see any doctor at all?

Yes ☐ → **GO TO Q21 (NEXT QUESTION)**

No ☐ → **GO TO Q22 (SKIP NEXT QUESTION)**

21 How many times has this happened in the past 12 months?

One time ☐ Two times ☐ 3 to 5 times ☐ More than 5 times ☐

Section 6. Experience of discrimination

22 In the last 12 months, have you ever been a victim of an ethnically motivated attack (verbal or physical abuse to you or your property) in New Zealand?

Yes, both verbal and physical ☐ Yes, physical only ☐

Yes, verbal only ☐ No, neither ☐

23 In the last 12 months, have you ever been treated unfairly (for example, kept waiting or treated differently) by a health professional (that is a doctor, nurse, dentist etc.) because of your ethnicity in New Zealand?

Yes ☐

No ☐

Not applicable – I have not visited a health professional in New Zealand in the last 12 months ☐

24 In the last 12 months, have you ever been treated unfairly at work because of your ethnicity in New Zealand?

Yes ☐

No ☐

Not applicable – I have not had a job or tried to find a job in New Zealand in the last 12 months ☐

25 In the last 12 months, have you ever been treated unfairly when renting or buying housing because of your ethnicity in New Zealand?

Yes ☐

No ☐

Not applicable – I have not tried to rent or buy a house in New Zealand in the last 12 months ☐

Section 7. Thank you and address for voucher

Research ID:

26 Thank you for completing this survey. A $20 Warehouse card will be posted to you in the next 2 weeks in recognition of your participation.

Tick this box if you would like the $20 card posted to the same address this survey pack was mailed to: ☐

Please write in your address below if you would like the $20 card posted to a different address than the one this survey pack was mailed to:

House / flat number: ☐

Street address: ☐

Town/Suburb: ☐

City: ☐

Postcode: ☐

Your personal information will only be used for mailing out your $20 Warehouse card. Your survey responses will remain confidential.

If you would like further information or advice about any of the health topics covered in the survey, you can contact one of the below helplines which are available 24 hours a day:

Helpline (for general health advice)	0800 611 116
Need to talk? Helpline (for mental health and addictions advice)	1737 (call or text)
Youthline	0800 376 633 (or text 234)
Lifeline	0800 543 354 (or text 4357)

Health and Disability Advocates (available Mon-Fri, 8.30am-5.00pm)	0800 555 050

Your usual doctor or other health professional can also provide advice if you have concerns about a particular health problem.

Please post your completed survey to Research New Zealand using the free-post envelope included in your survey pack.

Thank you for helping us with this important research.
